# Inheritance of fertility in broiler chickens

**DOI:** 10.1186/1297-9686-41-47

**Published:** 2009-10-29

**Authors:** Anna Wolc, Ian MS White, Victor E Olori, William G Hill

**Affiliations:** 1 Department of Genetics and Animal Breeding, Poznan University of Life Sciences, Wolynska 33, 60-637 Poznan, Poland; 2 Institute of Evolutionary Biology, University of Edinburgh, West Mains Road, Edinburgh, EH9 3JT, UK; 3 Aviagen Ltd, Newbridge, Midlothian, EH28 8SZ, UK

## Abstract

**Background:**

The fertility of a chicken's egg is a trait which depends on both the hen that lays the egg and on her mate. It is also known that fertility of an individual changes over the laying period.

**Methods:**

Longitudinal models including both random genetic and permanent environmental effects of both the female and her male mate were used to model the proportion of fertile eggs in a pedigree broiler population over the ages 29-54 weeks.

**Results:**

Both the male and the female contribute to variation in fertility. Estimates of heritability of weekly records were typically 7% for female and 10% for male contributions to fertility. Repeatability estimates ranged from 24 to 33%, respectively. The estimated genetic variance remained almost constant for both sexes over the laying period and the genetic correlations between different ages were close to 1.0. The permanent environment components increased substantially towards the end of the analyzed period, and correlations between permanent environment effects at different ages declined with increasing age difference The heritability of mean fertility over the whole laying period was estimated at 13% for females and 17% for males. A small positive correlation between genetic effects for male and female fertility was found.

**Conclusion:**

Opportunities to improve fertility in broiler stocks by selection on both sexes exist and should have an impact throughout the laying period.

## Background

Fertility is a trait of major interest in the broiler industry, primarily because of its effect on chick output. Also, as there is a negative correlation between growth and fertility, especially in naturally mated flocks [1], selection for growth alone over several generations is likely to result in a decline in fertility or in the ability of the males to mate efficiently [2,3]. Therefore, balanced selection should be aimed at improving key life performance traits while maintaining reproductive potential.

Fertility in poultry is traditionally regarded as an independent trait either of the male or the female, but genetic and non-genetic factors originating from both the male and female affect egg fertilization and embryo development [4]. Fertility of an individual egg is also a function of the genotype of the embryo, to which both parents contribute. Therefore, both paternal and maternal components should be accounted for simultaneously when analyzing fertility.

Factors affecting fertility which originate from the male include several sperm quality traits such as sperm metabolism, semen concentration, sperm motility, and the percentage of abnormal or dead sperm cells [5]. Behavioral factors include the male's ability to successively mate with the hens efficiently, which may be affected by leg problems [4] in the event of uncontrolled growth. Sperm quality traits are believed to be moderately heritable [6], whereas behavioral traits usually have a low heritability [7]. Factors originating from the female include egg quality, and behavioral and physiological factors such as prevalence of sperm storage tubules (SSTs) [4]. The issue of what is responsible for the decline in late fertility between sexes was recently addressed from a biological perspective by Barna [8]. In several studies, age has been shown to have a significant effect on fertility of broiler breeders [9-11]. Fertility generally declines after a peak [11] while the effect of age on female breeders is more significant than on male breeders [9,10]. The practice in a typical broiler breeding programme is to selectively improve traits within specialized male and female pure lines. To produce a commercial broiler, the aim is to enhance male fertility in a male line and female fertility in a female line with appropriate levels of selection pressure, but both components are important in line multiplication and in increasing selection intensity in nucleus populations. The traits of interest are the level of fertility and the ability to maintain high fertility over a long period, indicated by persistency. It is therefore highly desirable to provide an analysis of fertility which uses all available data to simultaneously estimate breeding values for male and female fertility. An analysis should account for all sources of variation, including the genetic and the permanent non-genetic environmental effects of the hen and her mate, and the effect of age and temporary environmental effects specific to each laying and hatching period.

Several approaches to analyze aspects of fertility in poultry can be found in the literature but none are all inclusive. For example, models used in recent analyses include fitting direct and maternal genetic effects for female fertility [12], fitting the trait as one of the females but also including male fertility in the model as a random non-genetic effect [13,14], which ignores the genetic contribution from the males, and longitudinal models including changes of fertility over time [15] based either on male or female fertility separately. Some have used likelihood or Bayesian approaches taking into account the binary nature of the trait [16], and one example of the desired model, which fits male and female components simultaneously, has been applied to time of lay in wild birds [17].

The different potential inputs into fertility, including sire, dam and offspring genotypes over a protracted period of life, provide questions about the genetic control of the trait which may have implications to other life history characters. Hence the present study was undertaken to investigate the various contributions to fertility by estimating genetic parameters for female and male fertility simultaneously using an animal model, taking into account the longitudinal nature of the trait by using random regression models.

## Materials and methods

### Data

Fertility data from a fully pedigreed pure broiler line were obtained from Aviagen Ltd, a primary broiler breeding company in the UK. The data comprised records of 555 males mated to 3755 females over a period of five years. Birds were housed in floor pens at a ratio of one male to ten females of the same age. The hens were trap nested with eggs set for incubation every week. Eggs were candled on the 7^th ^day of incubation to record the number of infertile (candled clear) eggs. Fertility was defined as the proportion of the total number of eggs set in each laying period that were fertile (as determined by candling). For the male, the total number of eggs set was the sum of all settable eggs laid by all the hens in the pen within the given period, while for the female, number of eggs set related only to the total number of settable eggs laid by the individual female within the period. Males were usually replaced if weekly fertility in a pen was consistently low for a period of five weeks. Data from such males were excluded from the analysis.

**Figure 1:**
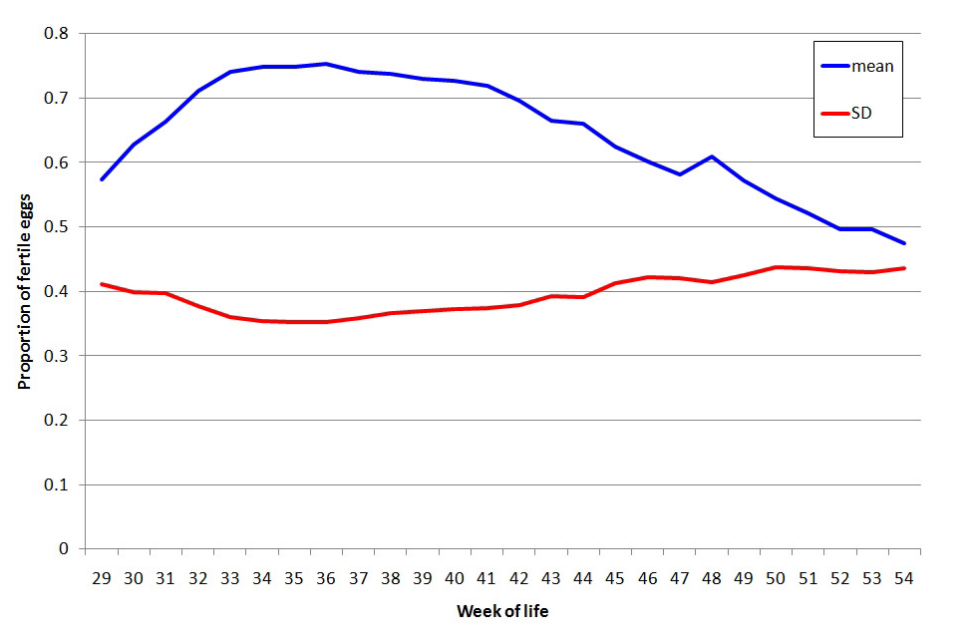
**Average fertility and its standard deviation for weekly records**.

The time trajectory used in this analysis was the age of the hen when the eggs were hatched. Records from ages 29 to 54 weeks were included, and comprised a total of 66104 weekly fertility records derived from a total of 342797 eggs set. The mean number of eggs set was 5.19 and, of the 66104 records, only 1557 were based on one egg. In the main analysis all weekly fertility records were used without weighting by the number of eggs set. The average weekly fertility across hens and over age is shown in Figure 1.

### Statistical analysis

All analyses were undertaken using ASREML [18], which permits fitting a sire-dam model with both components linked to the pedigree.

#### Random regression model fitting weeks

Fertility was described by the following random regression model using Legendre polynomials as covariates:

where:

*y*_*ijkn *_is a fertility record, i.e. the proportion of eggs set that were fertile, for incubation at the *n*-th week of age by female *k *mated to male *j *in POU *i*,

*POU*_*i *_is a fixed effect of contemporary group (which incorporates genetic trend),

*b*_*m *_is the *m*-th fixed regression coefficient,

*a*_M*jm *_and *a*_F*jkm *_are the *m*-th random regression coefficients for the additive genetic effect of, respectively, male *j *and female *k *mated to male *j*,

*p*_M*jm *_and *p*_F*jkm *_are the corresponding coefficients for permanent environmental (PE) effects,

*z*_*mn *_is the value of *m*-th degree Legendre polynomial at week *n*,

*e*_*ijkn *_is a random residual effect,

*m*_1 _to *m*_5 _are degrees of covariates fitted to fixed, genetic, and permanent environment effects, where *m *= 0 denotes a constant term and *m *= 1 a linear regression coefficient etc.

#### Choice of models

Since the use of random regression models fitting hen and mate genetic and permanent environment effects simultaneously for fertility of eggs is novel, it was necessary to consider a large array of models to determine which was the most plausible and provided an adequate description of the data. A subset of the many models fitted and corresponding likelihoods is shown in Table 1.

**Table 1:** Summary of effects^$ ^and degree of polynomials^# ^included in random regression (RRM) models (fitting weeks of lay), with corresponding log likelihoods (Log*L *relative to model 1)

Model	*a*_*m *_= *a*_*f*_^$^	*pe*_*m*_	*pe*_*f*_	Log*L*
Fitting repeatability model with constant error variance				
0	0	0	0	-3947.5
Fitting error variance for each 26 individual weeks of lay				
1	0	1	1	0
2	0	2	2	1357.7
3	0	3	2	1556.1
4	0	2	3	1786.5
5	0	3	3	1883.9
6	1	3	3	1888.9
Fitting genetic covariance of male and female effects				
5(+)	0	3	3	1884.1

^$^*a*_*m*_, *a*_*f *_genetic effects, *pe*_*m*_, *pe*_*f *_permanent environment effects for male and female fertility respectively

^#^Degree: 0 = constant, 1 = linear regression, 2 = quadratic regression, 3 = cubic regression

In the analyses presented, a cubic polynomial (*m *= 3) was used to model the fixed effect of age on fertility, as this described the observed mean weekly fertility profile (Figure 1). Increasing degrees of polynomials were tested to model the random permanent environment and genetic terms, and assessed by changes in the log likelihood. The likelihood increased substantially with increasing degree of polynomials fitted to the PE effects (shown up to cubic in Table 1), without reaching an apparent asymptote at quartic regressions. This corresponds with findings from other studies which suggest that increasing the order of fit for the random PE effects will continually increase the likelihood [19] (K. Meyer, personal communication). We considered a cubic regression to be a reasonable stopping point, after which the general shape of the relations of variances with weeks did not change qualitatively.

The residual variance, Var(*e*_*ijkn*_), was assumed to be different for each week of the age trajectory as fitting homogenous residuals over the whole or for three separate time segments gave much lower likelihoods, and we had no prior knowledge of the pattern of the error variances.

#### Covariance of male and female effects

In order to estimate the genetic correlation of male and female effects on fertility, a covariance between male and female genetic effects was also fitted in some models. This refinement was restricted to models with constant genetic effects (*m*_2 _= *m*_3 _= 0) in part because these models described the data almost as well as those of higher degree (see later), and in part because the simple models allow interpretation in terms of a single genetic correlation.

#### Threshold model

An unweighted analysis was used, but just simply weighting of weekly observations by the number of eggs set would not have been adequate because there was a high variance between parents in fertility. To accommodate both variation in numbers set per week and real differences between families, an analysis using a threshold model, which is much more computationally demanding, was undertaken fitting sire and dam genetic effects as constant (*m*_2 _= *m*_3 _= 0) and linear random regression PE terms (*m*_4 _= *m*_5 _= 1). However, it was not pursued further, because the relative size of all the genetic and PE components was found to be essentially the same as in the corresponding analysis using the unweighted model (data not shown).

## Results

The weekly proportion of fertile eggs was modelled as a trait of the hen which laid the eggs and of her mate, with both birds contributing genetic effects (structured proportional to the additive relationship matrix) and non-genetic individual specific (permanent environment, PE) effects. Variances of the random effects (genetic and PE) of both male and female on fertility were significantly greater than zero.

**Figure 2:**
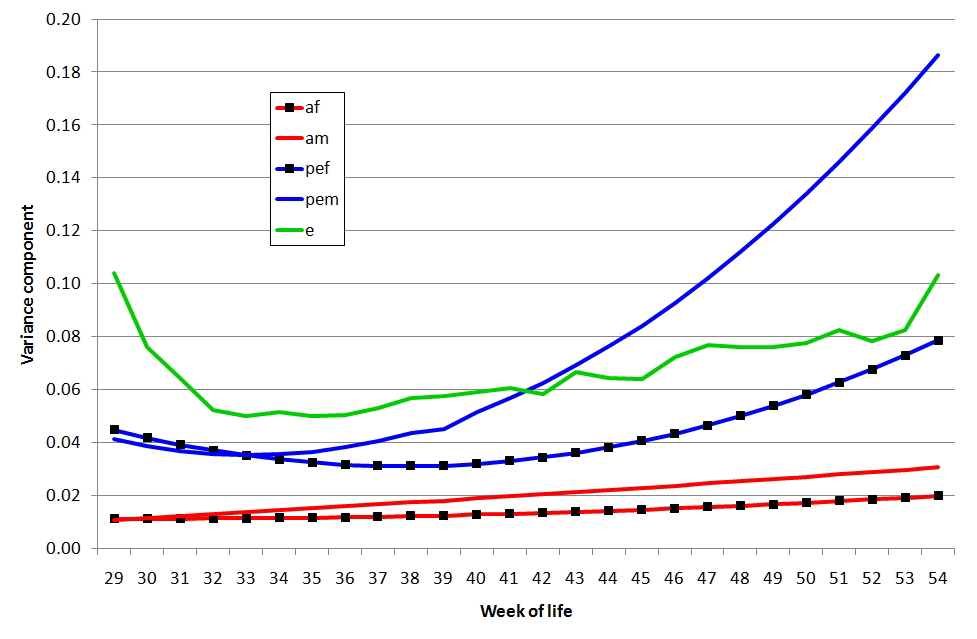
**Variance components estimated from linear random regression on week of age for genetic and permanent environmental components**. af = additive genetic effect of female, am = additive genetic effect of male, pef = permanent environmental effect of female, pem = permanent environmental effect of male, e = residual effect, with variance assumed heterogeneous over weeks

**Figure 3:**
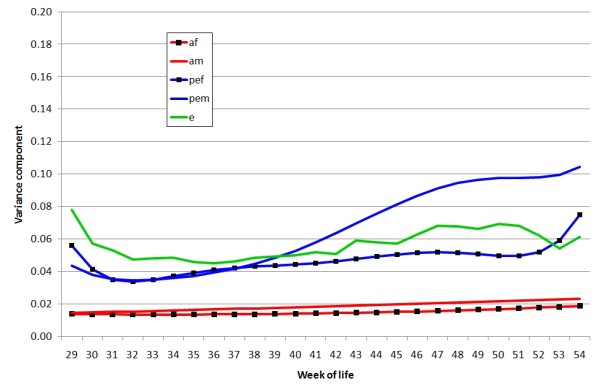
**Variance components estimated from linear random regression for additive genetic variance and third order random regression on week of age for permanent environmental components**. af = additive genetic effect of female, am = additive genetic effect of male, pef = permanent environmental effect of female, pem = permanent environmental effect of male, e = residual effect, with variance assumed heterogeneous over weeks

Variances due to non-genetic permanent environment effects of males (across mates and over time) and females (across time) were large and much higher than the corresponding genetic effects at all ages (Figures 2 and 3). These variance components increased non-linearly with age, with the male PE variance being higher than that of females. The difference between the magnitudes of the PE variances in Figures 2 (linear) and 3 (cubic) illustrate both that the variance contributed by PE effects, particularly of males, increases with age, but that the effects are less extreme than a linear regression would indicate. The increase in likelihood was small (approximately 5 for an extra 4 df) when the male and female genetic effects were modelled with a linear regression on weeks rather than a constant for each (Table 1) with almost all the increase due to females. Higher order genetic regressions were consequently not considered. Except where noted, the following results use a model with linear genetic and cubic PE terms.

**Figure 4:**
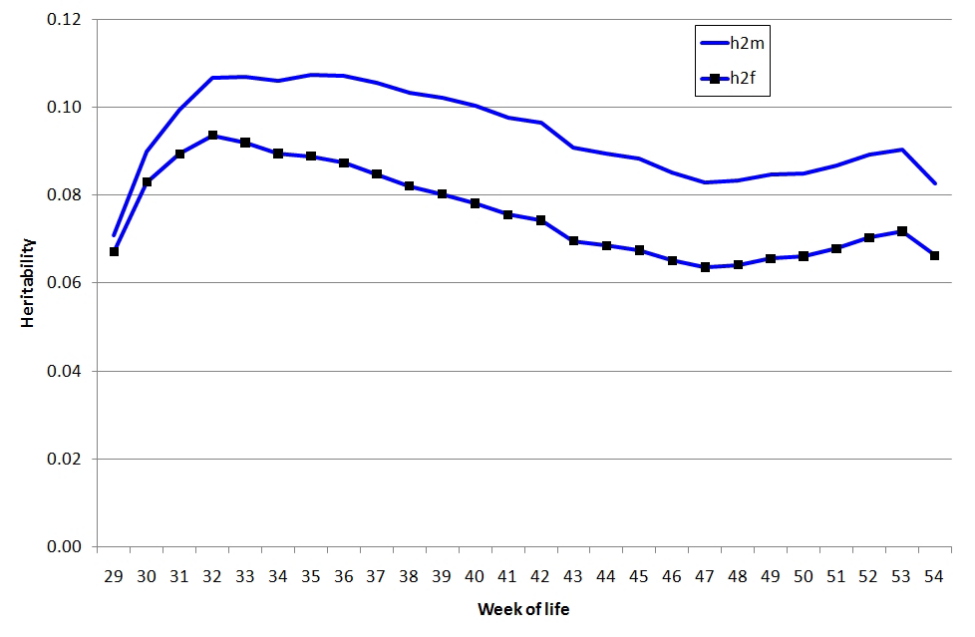
**Changes of heritability over age estimated from a random regression model as in Figure 3**. h2m = heritability of male fertility, h2 f = heritability of female fertility

The heritability for both male and female effects is plotted as a function of weeks in Figure 4. It was generally higher for males than for females, and did not change greatly with age, ranging from 7% to 9% for females and from 7% to 11% for males with the peak after a few weeks of laying.

Random regression analysis enables estimation of correlations between any pairs of age points. Examples of estimated genetic correlations and correlations of permanent environment effects for an early, mid and late week of production are given in Tables 2 and 3, respectively, in each case for both dams and sires. The genetic correlations for males are essentially one, and those for females exceed 0.7, even for the ages furthest apart (beginning and end of recording). This suggests that there is essentially no genetic variation in persistency of males and only a limited amount in females. The correlations of PE effects are much smaller, with a similar pattern in the two sexes, 50-80% for the more adjacent periods and nearer 20% for those furthest apart. These results indicate, in line with the graphs showing variances (Figure 3) that individual fertility changes substantially with age; indeed some birds become infertile.

**Table 2:** Genetic correlations between fertility at three different weeks of age of dams (below diagonal) and of sires (above diagonal)

		Sire		
	**Weeks**	30	40	50

	30		0.996	0.987
**Dam**	40	0.954		0.997
	50	0.839	0.955	

**Table 3:** Correlations of permanent environment effects between fertility at three different weeks of age of dams (below diagonal) and of sires (above diagonal)

		Sire		
	**Weeks**	30	40	50

	30		0.576	0.376
**Dam**	40	0.324		0.807
	50	0.262	0.582	

The inclusion of a genetic covariance between male and female genetic effects (fitted as constants) in the model did not result in a significant increase in the log likelihoods (Table 1). The estimate of the genetic correlation, 0.15, was consequently small, but was positive.

## Discussion

The present study shows clearly that both the female and her male mate influence the fertility of the egg, and that the contribution of each sex is influenced by both genetic and permanent environment components. Therefore, the male contribution is important, not only because each male is mated to many females but because, in a sire line, the individual male and his male descendents have a major influence on the efficiency of the multiplication pyramid. Furthermore, while the results pertain directly to domesticated poultry, there is no reason to doubt that there will be contributions of both sexes to egg fertility in natural populations of birds. However, obtaining adequate data to support or refute this conjecture in the wild would be difficult.

The conclusions are, of course, dependent to some extent on the model which is used for the analysis. We have not, for example, included maternal genetic effects because we considered it is more important to include the male genetic and permanent environment components when analyzing the trait as, essentially, one of the direct effects of the male and female mates, both of which had a large influence. With what are basically binary data, alternative analyses to cater for the heterogeneity in variance can be undertaken. For example a threshold model has been fitted in Bayesian analyses to data on fertility and other egg production traits in egg laying stock, but with the trait analysed solely as one of the female [16,20]. However, the permanent environment contribution of both sexes is large, such that the contribution from the binary variance taken over periods of weeks is relatively small, and also the change in the mean and thus phenotypic variance over ages is not great (Figure 1). We also fitted heterogeneous residual variances over the laying period to allow for any changes. Thus we consider that the analysis undertaken on the simple weekly proportion of eggs that were fertile, which is the trait definition use in the industry where hatching and selection occurs weekly, without weighting by numbers or scaling for the binary data is adequate and most relevant. Even so it may be worthwhile in future analyses to consider weighting by egg number or using longer time periods, or pursuing more complex analytical methods.

The random regression analysis appeared to show inflated values of the permanent environment terms towards the extremes of the laying period, particularly when fitting a linear regression for PE terms. Such a result is often considered a typical feature of the random regression model [21,22]; but it is most likely an indication that all sources of variation have not been adequately modelled, as indicated by changes when error variances were fitted with varying level of complexity [15,23]. It may also be due to the sparsity of records available at the extremes of the age trajectory, resulting in less information available to estimate the variances. Fitting higher order regressions reduced this extreme behaviour by more accurately partitioning total variance to all components.

The fertility in this line reached a peak, remained at a high level for some weeks and then slowly declined (Figure 1). Various factors can influence the decline in fertility with age, including yield of spermatozoa [24] and plasma composition [25] of the male and the efficiency of sperm storage of the female [10]. Although early studies have indicated that in most cases males are responsible for completely infertile matings and that replacing them leads to fertility in over 90% of matings [26], more recently the role of females in persistency of fertility has been stressed [8,27]. Fertility in females has been found to be more susceptible to the influence of age than that of males [9,10].

Our study shows that the genetic correlation between fertility at different ages for both males and females was very high across ages and was close to one for the male component (Table 2). The contributions from permanent environment effects in both sexes increased with age, particularly for males (Figures 2 and 3) and were much larger than those from the genetic effects, but the correlations across ages were much lower. This pattern agrees with a finding that males having a poor sperm quality index at a young age have a low sperm quality subsequently and a large decline in fertility after the peak [28].

The heritability of fertility estimated in this study was low at all ages. This is not surprising as it is a fitness related trait. Both male and female components increased initially to a peak, at about 9% for the female and 11% for the male, and then declined with age (Figure 4). For comparison with other reports, heritability was also estimated using a repeatability model in which the genetic and permanent environmental variances were assumed to be constant over ages. With this model the estimates of heritability for one week's set of eggs averaged over the laying period were 7% for females and 10% for males and those for repeatability were 24% and 33%, respectively. The magnitude of these estimates falls within the range found in the literature for female fertility. For example, Sapp et al. [13] analysed female fertility with a repeatability model and reported heritability estimates ranging from 5.5% to 7.4% depending on the way in which missing values were treated. Similar estimates were obtained for liability of fertility (*h*^2 ^= 6.7%; repeatability = 22%) using a threshold Bayesian model [16].

Higher heritability and accuracy of selection may be obtained by averaging fertility over several weeks of set, either by pooling over eggs or by averaging weekly fertilities. However, in view of the high PE variances, the increase in heritability is not great. For the simple average of weekly fertility records, we predict it would reach 13% for females and 17% for males. Presumably, if all records were pooled with appropriate weightings and corrections for week of laying effect, somewhat higher values would be predicted. The genetic correlation between ages was high for both sexes, particularly males, which indicates that early records of fertility can be used to make breeding decisions and that there are limited opportunities to specifically improve fertility in older flocks, which is when production is most limited. Even so, basing selection decision on records from the early stages alone, gives no emphasis to the important issue of persistent fertility as the flock ages.

The genetic correlation between male and female fertility used here refers essentially to the correlation between the fertility of eggs produced by daughters and by the mates of sons. The correlation was positive in our study but moderate and non-significant (0.15), indicating that improvement of one sex would be slightly beneficial, and certainly not harmful to the other, but that selection on records of both sexes would be needed for maximum improvement of overall flock performance. This could also be achieved by fitting a sire-dam multivariate BLUP model using cumulative records during the laying period from which breeding values for fertility of both sexes can be obtained for each candidate for selection.

Several interesting questions remain for future research. One question is the magnitude of the genetic variance of hatchability after candling at 7 days, and its correlation with (pre-candling) fertility. Another one is the correlation between fertility and performance traits such as growth rate, although the analysis is not straightforward as only selected birds have the opportunity to breed. This also implies that, if the correlations are high, there may be biases in the current estimates of genetic variances and heritability as the data have, in effect, been censored. As this study shows, such analyses have to cater jointly for effects of both sexes and, for the reproduction traits, incorporate both genetic and permanent environment effects.

## Competing interests

The authors declare that they have no competing interests.

## Authors' contributions

All authors conceived the study, contributed to methods and to writing the paper and also read and approved the final manuscript. AW undertook the analysis and wrote the first draft. Data from Aviagen were prepared by VEO.
